# From Plant Compounds to Botanicals and Back: A Current Snapshot

**DOI:** 10.3390/molecules23081844

**Published:** 2018-07-24

**Authors:** Alessandra Durazzo, Laura D’Addezio, Emanuela Camilli, Raffaela Piccinelli, Aida Turrini, Luisa Marletta, Stefania Marconi, Massimo Lucarini, Silvia Lisciani, Paolo Gabrielli, Loretta Gambelli, Altero Aguzzi, Stefania Sette

**Affiliations:** CREA-Research Centre for Food and Nutrition, 00178 Rome, Italy; laura.daddezio@crea.gov.it (L.D.); emanuela.camilli@crea.gov.it (E.C.); raffaela.piccinelli@crea.gov.it (R.P.); luisa.marletta@crea.gov.it (L.M.); stefania.marconi@crea.gov.it (S.M.); massimo.lucarini@crea.gov.it (M.L.); silvia.lisciani@crea.gov.it (S.L.); paolo.gabrielli@crea.gov.it (P.G.); loretta.gambelli@crea.gov.it (L.G.); altero.aguzzi@crea.gov.it (A.A.); stefania.sette@crea.gov.it (S.S.)

**Keywords:** dietary supplements, botanicals, bioactive compounds, antioxidants, study approach, integrated food research, dedicated databases, dietary assessment.

## Abstract

This work aims at giving an updated picture of the strict interaction between main plant biologically active compounds and botanicals. The main features of the emerging class of dietary supplements, the botanicals, are highlighted. Focus is also on the definition of actual possibilities of study approach and research strategies. Examples of innovative directions are given: assessment of interaction of bioactive compounds, chemometrics and the new goal of biorefineries. Current models of existing databases, such as plant metabolic pathways, food composition, bioactive compounds, dietary supplements, and dietary markers, are described as usable tools for health research. The need for categorization of botanicals as well as for the implementation of specific and dedicated databases emerged, based on both analytical data and collected data taken from literature throughout a harmonized and standardized approach for the evaluation of an adequate dietary intake.

## 1. The Emerging Class of Dietary Supplements: A Mini Overview of Botanicals Features

The field of food supplements appears varied and growing: a wider spectrum of new products appears on the market every year. This reflects a new reorganization of the market for dietary supplements, resulting from new strategies, technologies and also the changes in the regulation applied to nutrition and to health claims. The growth of this sector is encouraged by greater consumer interest in improving physical and mental wellbeing and health status, often to compensate for an incorrect lifestyle [1,2,3]; as reported by Ekor, [4], over four billion people of the world’s population use herbal supplements as products of medical care; there is a great and widespread growth in the consumption of herbal remedies [5], related generally to their easy availability, but also to the easy and false perception or idea, that many people feel that what is “natural” is supposed to be healthful and safety, never toxic or side effects.

With the term botanicals is here indicated herbal remedies, herbal drugs, herbal medicinal products, herbal medicines, botanical drugs as synonyms; numerous are the definitions of the term botanicals in relation to the different fields (i.e., pharmacy, botany, medicine, nutrition) [6,7,8]. Herbal medicinal products are referred to “any medicinal product, exclusively containing as active ingredients one or more herbal substances or one or more herbal preparations, or one or more such herbal substances in combination with one or more such herbal preparations” as introduced in 2004 by Directive 2004/24/EC of the European Parliament and of the Council of 31 March 2004 [9]. 

Here the definition by the World Health Organization (WHO) is also reported as follows “Herbal medicines include herbs, herbal materials, herbal preparations and finished herbal products, that contain as active ingredients parts of plants, or other plant materials, or combinations” [10].

Botanicals are made of single herbs or by mixing different herbs, from raw material of whole plants or parts of them, and include flowering herbs, leaves, leaf exudate, fruits, berries, roots, rhizomes, fungi, microorganisms, algae.

Starting from these materials, numerous and different techniques and procedures i.e., extraction, distillation, purification, fractionation, concentration, fermentation, etc. are used to obtain botanical substances (single active compounds or more compounds of a chemical class) and preparations/formulation (i.e., extracts, tinctures, powders). Botanicals are prepared through a complex, specific and detailed procedure of preparation process, and on this matter reference and regulation books are available [6,11,12,13].

In this regard, as described by Alamgir, [14], in a recent 2017 work, it is important to distinguish between pharmacopoeia reference books for the preparation of quality medicines, and herbal and therapeutic compendium, an accurate description of botanicals, as Materia Medica [14].

Moreover, the WHO has developed a portal [15], that contains 5845 medicines and health product-related publications taken from WHO, other United Nations (UN) partners, global Non-Governmental Organizations (NGOs), development agencies and their partners, countries and academics, and is updated monthly [16]. It is important to mention the guidance document formulated by the European Food Safety Authority (EFSA) with a science-based approach, on how to assess the safety of botanicals and of botanical preparations/formulations to be used in the food sector; in particular, a list of the main categories of botanicals and safe botanical preparations/formulations was established [6,7]. 

The recent study of Breemen et al. [17] summarized and well described the main steps for the development of botanical dietary supplements, underlying how these steps should be similar to those of pharmaceuticals: definition of action mechanism of main bioactive compounds, chemical standardization related to the main compounds and biological standardization linked to pharmacological activity, bioavailability studies, toxicity evaluation, preclinical evaluation, clinical studies of safety and efficacy.

## 2. Study Approach about Botanicals and Their Main Plant Compounds: Up-to-Date, Current and Innovative Directions

In this paragraph examples of actual possibilities and innovative directions of research strategy are given: assessment of interaction of bioactive compounds; chemometrics; new goal of biorefinery.

### 2.1. Main Plant Compounds and Their Interactions Assessment

Plants are the source of a magnificent spectrum of compounds and, in this order, are defined as one of the most efficient chemical systems known [18]: 200,000–1,000,000 different metabolites are estimated to be synthetized in the plant kingdom [18].

The diversity of plant compounds derived from the infinite combinations of fundamental functional groups such as hydroxyls, alcohols, aldehydes, alkyls, benzyl rings, steroids that originate compounds with peculiar chemical and physical characteristics (i.e., solubility, melting point, and reactivity) [19]. 

The combined and concerted action of phytochemicals, (i.e., polyphenols, carotenoids, glucosinolates, lignans, etc.) gives the potential beneficial properties of each plant matrix [20,21]; these concerted interactions are responsible for a large spectra of physiological and biological functions (i.e., anti-inflammatory, antioxidant, anti-allergic, antimicrobial, anti-atherogenic, etc.) [22]. Many original researches and reviews are present in literature on the relationship compound-activity [23,24] as well as chemical class and related bioactivity, i.e., alkaloids [25,26], saponins [27], terpenoids [28], polyphenols [29,30], etc.

As defined by Biesalski et al. [31], “bioactive compounds” are compounds that occur in nature, part of the food chain, that has the ability to interact with one or more compounds of the living tissue, by showing an effect on human health.

The identification, isolation and quantification of bioactive compounds as well as the assessment of their interactions, in the specific case the definition of herb-drug ones, could be considered as the main steps in the study of the potential beneficial plants’ properties.

In this regard, it is important to underline how the quantification of bioactive compounds present in the plant extracts is required as starting point. This output involves the applications of a large spectra of techniques as described by several authors [32,33,34,35]. As instance, Sasidharan et al. 2010 [32] summarized the main analytical techniques for the ingredient characterization in herbal preparations, by highlighting the relevance of extraction procedures. Ingle et al. [34] categorized in a detailed manner the main techniques of extraction and analysis for botanicals. Ganzera et al. [33] focused on recent perspectives and application in botanicals of HPLC/MS. Pandey and Tripathi, [35] defined and exploited the standardization in drug analysis.

The concerted actions of compounds bioactivity and the related activities of food extracts, were clearly studied and discussed by Durazzo, [20]; the author underlined how two complementary approaches can be applied: either the evaluation of bioactivities of pure compounds and/or their mixtures or the isolation of different biologically active compound-rich extracts and how these fractions contribute to the total activity of food extract.

### 2.2. Integrated Research, Emerging Technologies and Chemometrics

Among recent approaches with rapid and green procedures, direct analysis such as fluorescence, near infrared (NIR), mid infrared (MIR), nuclear magnetic resonance (NMR) spectroscopies, infrared spectroscopy, multi-elemental analysis, isotopic ratio mass spectrometry, etc. produce large datasets representing the input for multivariate data analysis methods. Therefore, studies on the evaluation of bioactive components are generally integrated into a multidisciplinary system of detection and analysis, generating data matrices for the application of statistical methods like in the chemometrics science.

Moving from bioactive components to botanicals, chemometrics opened a new scenario for herbal drugs [36]; the chemometric approach represents a valid tool in the following actions: authentication of individual herbs, monitoring of the quality of herbs and herb medicines, identification of chemical constituents, detection of adulteration or contamination of herbs, production of standardized formulations. 

In the past, two were the main approaches for quality control of herbal medicines, the ‘component-based’ and ‘pattern-based’ ones [37,38]. The first approach was focused on the study of specific compounds with defined properties (i.e., marker approach and multi-compound approach), whereas the latter one studied all detectable compounds (i.e., the pattern approach and the multi-pattern approach). 

Considering the complexity of herbal ingredients used for testing, the markers do not allow an adequate evaluation of the quality assurance of the herbal materials in all cases; generally, one or two markers are necessary for quality control and authenticity of herbal medicines. The lack of a unique marker did not allow a total overview of an herbal product representing a real problem when qualitatively differentiating them. 

An emerging intervention strategy is given by the fingerprint analysis and chemometric technique that make feasible the comparison of compositions in nutrients and bioactive compounds in numerous and different samples, i.e., using all the components detected through their whole chromatograms acquired from spectroscopy, liquid chromatography, gas chromatography, mass spectrometry, and so on. Indeed, this new approach allows the workflow for chemistry, manufacturing, the quality assessment and controls of botanical drugs. In this regard, the recent work of Harnly et al. [39] provided an overview on how to detect the transition of chemical composition from botanical ingredients to resulting products by using chemometrics, differentiating them quickly.

In this context, the identification and formulation of innovative types of quality markers is required and, in this direction, multi-compound, multi-target and multi-pathway studies are being carried out. 

In a recent work, Yang et al. [40] proposed the use of bioactive chemical markers, a cluster of chemo-markers showing similar pharmacological activities and comparable to the whole botanical drug: bioactive chemical markers based strategy was formulated and applied to *Xuesaitong Injection*. 

As another example in this direction, Zhang et al. [41] gave a general prototype combining the chromatographic fingerprint of bioactive compounds and bioactivity assay to elucidate the relationship spectrum–effect, in a traditional Chinese plant, *Acalypha australis* Linn. 

Also Abubakar et al. [42] in an effective research have proposed and discussed DNA barcoding in combination with chromatography fingerprints for the authentication of botanical ingredients in herbal medicines.

The current review of Sánchez-Vidaña et al. [43] emphasized how novel advanced technologies in the field of traditional Chinese medicine research are required for the implementation of separation methods, standardization techniques, quality control, the understanding of the action mechanism of single compounds, clinical validation assays; in this order, the application of omic technologies represents a promising approach in phytotherapy [38,39,43,44].

### 2.3. Food Waste as Source of Bioactive Compounds: A New Goal of Circular Bioeconomy and Biorefinery

Another innovative direction concerning the botanicals research is given by the diffusion of use of food waste as a sustainable alternative source of biologically active compounds. The “Universal Recovery Strategy” for the commercial recapture of valuable compounds from food wastes is a new goal of the circular bioeconomy and the biorefinery concept [45,46,47,48,49]. The bioactive compounds are nowadays recycled inside food chain from field to fork [50,51]: they are extracted, recovered and reutilized from food byproducts to formulate functional foods and nutraceuticals [52]. The agro-industrial field gives a great opportunity when considering the large quantities of waste and by-products generated every year in the processing of fruit and vegetables. In particular, by-products of plant food processing represent a promising source of biologically active compounds, which may be used for their favorable technological or biological properties; moreover, the use of new technologies is utilized to reinforce and increase the “Green Economy” in agriculture and agro-industry [53,54].

It is worth mentioning the work of Pfaltzgraff et al. [55] that described and schematized well the components (i.e., pectin, sugars, starch, collagen, amino acids, polyphenols) present in food supply chain residues (i.e., tomato pomace, wheat straw, rice husks, spent Brewer’s grain) and their uses in common consumer applications. Another interesting review is the work of Baiano et al. [56], that gave detailed and updated description of the type and amounts of food wastes and their legislation as well as conventional and innovative techniques for the extraction of bioactive compounds; also the future trends in nutraceutical, cosmetic, pharmaceutical sectors were discovered [56].

## 3. From Metabolic Pathways to Bioactive Compound Databases: Tools towards Health

Current models of specialized databases represent effective tools to study the relationship between plant natural compounds and botanicals. In this paragraph, an updated description and a shot of the current state are given and discussed: plant metabolic pathways databases; food composition databases; bioactive compound databases; dietary supplements databases; dietary markers databases. Several and different tools are being developed for secondary metabolic pathways, biological activities, chemical structures, ethnobotanical uses, content in foods, and pharmacology; they represent open source and queryable that can serve as updated sources of information [57].

As starting point, recent examples of plant metabolic pathway tools are given by KEGG Bioinformatics Resource [58]—a tool for interconnection between Plant Genomics and Metabolomics data—and by Plant Reactome [59]—a database that gives bioinformatics tools for visualization, analysis and interpretation of plant metabolic pathways—all aiming to support modelling, systems biology, genome annotation and analysis, basic research and education. 

Moving into the nutrition scenario, food composition databases represent the main tools for numerous interventions: elaboration of food consumption data, allowing to convert them into nutrient intake; evaluation of the nutritional and health status of a population; carrying out of epidemiological studies; formulation of diets at the individual and/or population level; epidemiological and clinical research; nutritional education; support industrial and handicraft companies for the labeling [60,61,62]. The European Food Information Resource (EuroFIR) Network of Excellence and Nexus projects (2005–2013) put the basis of harmonization of Food Composition Databases through standardized protocols and food description system i.e., LanguaL™ [63]. The EuroFIR-AISBL, an international non-profit Association, provides a widespread resource at European level for compilers and users of food composition data throughout a large set of online tools, i.e., FoodEXplorer, Food Basket, eBAsis, PlantaLibra [64]. FoodEXplorer online tool is a virtual platform that combines 30 national standardized and specialized food composition databases (Europe, the United States, Canada Australia, Japan), including more than 40,000 foods [60]. The International Network of Food Data Systems (INFOODS) by FAO, contributes to improve the accessibility, reliability and management of food composition data at worldwide level [65].

For bioactive compounds, the major public, core comprehensive databases are: the United States Department of Agriculture (USDA) databases [66]; the Phenol-Explorer database [67,68]; Bioactive Substances in Food Information Systems (eBASIS) [69,70,71]; ePlantLIBRA database [72].

The USDA database was developed in 2004 and it is based on a compilation of data from literature and expanded in recent years to include flavonoids, proanthocyanidins and isoflavones [73,74,75]. 

Phenol-Explorer represents the first comprehensive open access database on content of polyphenols in foods; several updates on pharmacokinetic and metabolites, effect of food processing and cooking were carried out [76,77]. Five steps—literature search, data compilation, data evaluation, data aggregation, data exportation to the MySQL database (used by the web interface)—were carried out during the procedure of development of the Phenol-Explorer database. Composition data were taken from the peer-reviewed scientific publications and evaluated, then they were aggregated to obtain mean values. 

The eBASIS, the first EU harmonized database, combines composition data and biological effects on over 300 major European plant foods of 24 compound classes, e.g., glucosinolates, phytosterols, polyphenols, isoflavones, glycoalkaloids, xanthine alkaloids [70,71]. EuroFIR eBASIS resource represents a collection of data from peer-reviewed literature evaluated critically by experts and inserted as raw data. 

Nowadays, there is the need to include extractable and non-extractable phytochemicals in bioactive compounds databases [78] for a better dietary intake assessment: extractable compounds are those that are present in free forms and are solubilized by aqueous–organic solvents, whereas non-extractable compounds are bound forms, remaining in the residue of aqueous-organic extract. In this direction, development and expansion of eBasis structure was addressed [79].

Concerning the specialties of food supplements, ePlantLIBRA database [80], developed within the PlantLIBRA (PLANT food supplements: Levels of Intake, Benefit and Risk Assessment) project, represents a comprehensive and searchable database, with up-to-date information on bioactive compounds specific for plant food supplements, reporting health benefits, adverse effects, contaminants and residues. In order to search, extract, and export the data, ePlantLIBRA database was structured as user-friendly, efficient and flexible interface, by including also links to the original references [72]. As reported by Plumb et al. [80] in ePlantLIBRA, data from over 570 publications have been evaluated for quality, by covering seventy plant food supplements or their botanical ingredients.

Considering the importance of dietary supplements in the evaluation of dietary intake, as revealed during the National Health and Nutrition Examination Survey, NHANES, a dietary supplement label database [81,82] was developed and launched in 2013 by the Academy of Nutrition and Dietetics in the United States: now it contains supplement label information (brand name, ingredients, amount per serving, and manufacturer contact information) of about 50,000 dietary supplements present in the U.S. marketplace and consumed [11,83]. Browsing options were developed and organized to search by product, ingredient or contact of manufacturer, by representing a useful tool for consumers, professionals, researchers [83,84].

In this context, recently, at European level, within the PD Manager Project [85], information on the composition of dietary supplements taken from labels, and according to the Italian market were collected and updated for the development of a Dietary Supplement Label Database [86]: 212 items were inserted, by trying to give a uniform and representative picture of the main classes of dietary supplements consumed in Italy, and 82 descriptors were included, in addition to nutritional information: Brand name, Food group, Distributor, Producer, Packaging sizes, Unit weights, Data source, Additional remarks [86]. It is important to underline that for each item a code was assigned following the food classification system FoodEx2 developed by EFSA [87], to allow the standardization and harmonization of data among different countries; feedbacks and proposal for FoodEx2 revision 2 implementation, with focus on dietary supplements, [88] as well as a constant update and implementation of Dietary Supplement Label Database are ongoing [89].

At the same time the understanding of activities and benefits of bioactive compounds in humans is essential; however, the evidence derived from human intervention gave limited and conflicting results, partly due to differences in absorption, distribution, metabolism and excretion between individuals [90]. The recent work by Dragsted et al. [91] underlined the importance of databases for dietary biomarkers for the main food groups and new data on non-nutrients compounds and their metabolites. The Human Metabolome Database or HMDB 4.0 [92] is a web metabolomic database on human metabolites [93]. PhytoHub is a freely electronic database containing detailed information about all phytochemicals commonly ingested with diet and their metabolites [94,95].

## 4. Conclusions

During the last decade botanicals, the newest class of dietary supplements, have emerged and their use is spreading among consumers, although they should not replace a correct lifestyle and/or a healthy diet. The scientific community is addressing towards the development and assessment of methodologies to isolate and standardize fractions with specified bioactivities from medicinal plants. Emerging technologies combined with chemometrics are being applied to medicinal plants in an innovative and integrated research approach, also in the directions of circular economy and biorefineries.

In addition, studies on the description and exploitation of bioactive compounds in medicinal plants as well as on physiological mechanism and bioaccessibility of compounds are being carried out. 

The overall goal is the categorization and classification of botanicals as well as the development and implementation of dedicated databases, based on specific analytical and collected data, and achieved throughout a harmonized and standardized approach in order to evaluate a correct dietary intake.

It is important to underline how nowadays the “botanicals” class is expanding from herbs and medicinal plants to also include some foods, i.e., artichoke, garlic, etc. This suggests enhancing the value of foods by also investigating their functional/nutraceutical characteristics, in order to integrate intrinsic nutritional properties.
